# Motivating green behaviour in Bangladeshi employees: Self-determination theory application

**DOI:** 10.1016/j.heliyon.2023.e18155

**Published:** 2023-07-13

**Authors:** Abdullah Mohammad Ahshanul Mamun

**Affiliations:** Department of Business Administration, International Islamic University Chittagong, Sitakundo, Kumira, Chittagong, 4318, Bangladesh

**Keywords:** Self-determination theory, Employee green attitude, Employee green motivation, Employee green behaviour, Sustainability

## Abstract

Academic research indicates that Bangladesh has relatively low levels of employee green behaviour. Developed and developing nations worldwide have raised environmental awareness within their workforces in response to the alarming rate of environmental deterioration caused by human activities. This study looks into potential links between the motives (intrinsic and extrinsic), green behaviour, and attitudes of Bangladeshi employees. A theoretically supported framework has been developed using self-determination theory. Purposive sampling was used for this survey on Bangladeshi financial institutions (private banks). Data were analyzed using partial least squares based structural equation modeling (PLS-SEM). The findings propose that employee green attitude mediates the relationship between intrinsic motivation and employee green behaviour. Employee green attitude (EGA) also mediates the relationship between employee green behaviour (EGB) and introjected, identified, integrated, and external regulation (external motivation). The outcomes display a negative relationship between introjected regulation and employee green behaviour as well as external regulation and employee green behaviour, contradicting other research. In Bangladesh, EGA and EGB are understudied; therefore, this research has significance. This research will assist the international community in addressing environmental, social, and economic concerns for a more sustainable future.

## 1 Introduction

Organizations in both industrialized and emerging countries are under increased pressure to hire environmentally concerned employees because of extensive environmental deterioration brought on by human activity [1]. Businesses should encourage employees to adopt environmentally friendly policies and processes in order to safeguard the planet's ecosystem and natural resources. A conscious effort by an employee to reduce the effects of human actions on the environment is characterized as “employee green behaviour” [2]. It may be recognized as employee behaviours favourably influencing the environment [3], such as recycling, effective resource usage, water conservation, decreasing trash, Etc [4]. Moreover, in the twenty-first century, environmental sustainability is increasingly important for corporate survival [5], and many firms prioritize environmental sustainability, reflecting community preference [6]. Besides, the goals and advantages of environmental sustainability must be widely mentioned in corporate events with employees [7]. Nevertheless, researchers have noticed a mismatch between corporate sustainability attributes and human resource management practices [8]. Specifically, performance management systems that expressly motivate employees to support organizational sustainability initiatives are necessary [9]. In this regard, employees' green behaviour has been considered to be a form of pro-environmental conduct specific to the organization by researchers [10]. To motivate employee green behaviour, firms must focus on institutional support, organizational leadership, and peer factors [11]. Even though research indicates that employees are aware of their employer's environmental commitment and how their individual and professional environmental actions interact [12], however, the majority of research conducted in this field focused on creating organizational policies and rules for environmental protection [13], which may not convert into the actual environmental behaviour of employees for several reasons [14].

The influence of human resource management on environmental sustainability is crucial for achieving stable business growth, and studies have highlighted the need to implement environmental sustainability metrics into the human resource management system [15]. Employees with a favourable attitude toward sustainability are more inclined to take actions that help the environment and exhibit greater green motivation [16]. Furthermore, organizations encouraging sustainability and allowing employees to participate in green activities have been reported to boost employee engagement and job satisfaction [17]. Thus, firms should emphasize the development of a culture of sustainability to encourage good employee attitudes, motivation, and actions toward the environment. Although academia has experienced numerous research studies conducted focusing on “Pro-environmental behaviour ", “green behaviour”, “environment-friendly behaviour,” or “low-carbon behaviour” [18], norms and environmental behaviour [19], workplace drivers [12], environmental regulation [13], green human resource management practices [20] no such studies paid attention on employee green attitude as a mediator between green motivation and green behaviour based on self-determination theory so far the authors best knowledge. However, a few studies generally investigated employee green attitude role in determining the relationship or impact of job satisfaction [16] and green work climate perception [17]. In particular, very few papers concentrated on specific factors of employee green motivation [21], such as intrinsic motivation, identified regulation, and external regulation. According to the author's information, this is the initial empirical study focusing on the use of employee green motivation and employee green behaviour on the employee green attitude of Bangladeshi employees.

This study is backed by the self-determination theory of Deci and Ryan, which describe the motivation of individuals into two types: Intrinsic motivation and extrinsic motivation, whereas extrinsic motivation is further classified as introjected regulation, identified regulation, integrated regulation and external regulation [22,23]. The present study aims to examine the potential mediating role of employee green attitude in the association between employee green motivation and employee green behaviour. Specifically, the study will explore whether employee green attitude mediates the relationship between employee green motivation, consisting of five variables: intrinsic motivation, introjected regulation, identified regulation, integrated regulation, external regulation, and employee green behaviour.

It is evident from the discussion that research into the voids in our knowledge on the motivation of employee green behaviour is warranted, especially in the context of Bangladesh as a developing economy. There is a need for academic study on the elements that contribute to developing a caring work culture that values environmental sustainability and encourages its members to take environmentally responsible actions. There is a need for more recent research on employee green attitude and employee green behaviour, and this study fills that vacuum by focusing on Bangladesh. Such studies will benefit a global audience by equipping them with tools to address environmental, social, and economic challenges and build a more sustainable future.

This study contains seven sections. Section two will discuss the previous literature and review self-determination theory. Section three will discuss hypothesis development. Section four will elucidate the research method and data. The results and discussion will be represented in the fifth and sixth sections, respectively. Section seven describes the research conclusion, theoretical and managerial implications of the study, and the study's shortcomings as well as future directions. The study's findings add to our knowledge of the factors influencing employees' intentions to do environmentally friendly behaviours and have implications for academicians and public policymakers locally and globally.

## 2 Previous literature

Human activity is responsible for rising pollution and environmental destruction [24]. Moreover, over recent years, these effects have seriously harmed the Earth's carrying capacity [24]. Employees, for instance, can save energy by turning off electrical devices, walking by the stairway instead the elevator, doing both-sided paper printing, reducing trash, and brainstorming thoughts for environmental conservation [25]. Environmental protection through individual actions is stated as “Pro-environmental behaviour (PEB)", “green behaviour”, “environment-friendly behaviour”, or “low-carbon behaviour” [18]. PEB is a combination of environmental obligations that include enhancing environmental knowledge, developing environmentally friendly goods and procedures, and evaluating environmentally destructive behaviour [26]. PEB has been divided into two categories [2,18]: personal PEB (such as purchasing, utilizing, and discarding own products and services) and community PEB (e.g., environment conservation regulations, motivating citizens to participate in green actions, and tackling environmental matters). The researchers discovered that economic development, overseas investment, local investment, urban growth, infrastructure, and quality of institutions all positively impact sustainable energy and non-renewable energy demand in Bangladesh [27], and the nation still needs more energy security [28]. Although environmental policies, laws, and regulations exist for various service industries, many workforces must be aware of them. Particularly at higher management levels, 'caring for the environment’ is less significant to formal and informal workforces [29].

Although Bangladesh has much potential, it needs more information and technical expertise to utilize renewable energy sources effectively. The use of renewable energy technology could provide an alternative method of satisfying Bangladesh's expanding energy needs [30]. Organizational citizenship behaviour toward the environment (OCBE) was positively benefited by sustainability education and training, sustainability-focused assessment methods, and employee autonomy [31]. Both cognitive and non-cognitive factors, such as - fostering sustainable workplace guidelines and applications, can improve employees' task-related EGB [32]. This research recommends a new research viewpoint that reflects individual motivational factors to gain more attention to the problem.

The SDT is a broad supposition of individual expansion and growth that emphasizes the interaction between the energetic, development-focused personality and the community atmosphere [22,23]. It encompasses *intrinsic motivation*, involving activities naturally exciting or rewarding, and *extrinsic motivation*, which entails following actions due to external factors [23]. Ethical standards and injunctive moral values positively influence intrinsic and extrinsic pro-environmental behaviour among employees [33]. Extrinsic motivation can coexist with intrinsic motivation. When people are motivated autonomously, they seek actions congruent with their basic identities [23]. Deci and Ryan established a classification of extrinsic motivation that includes four types of regulations: introjected, integrated, identified, and external. Employees' self-identity, including both extrinsic and intrinsic extent, might considerably impact their motivations, approaches, and green behaviour [22]. Researchers have come to recognize the value of a green self-identity in influencing employees' decisions to act ecologically friendly manner on account of the idea of self-determination. These factors led to using SDT in this study to examine relationships between specific motives, attitudes, and environmentally friendly behaviour. More research on SDT is needed to elucidate employee green motivation. While Kim et al. (2022) used SDT to improve their comprehension of the psychological phenomenon associated with becoming an environmental activist in the organization [34], Dodds et al. implemented the drivers of the sustainability framework. They connected it to the psychological factors mentioned in SDT in the setting of Canadian festivals [35]. So, from the discussion above, there is a knowledge gap in understanding the drivers behind employee green behaviour at the workplace.

## 3 Development of hypotheses

**Fig. 1:**
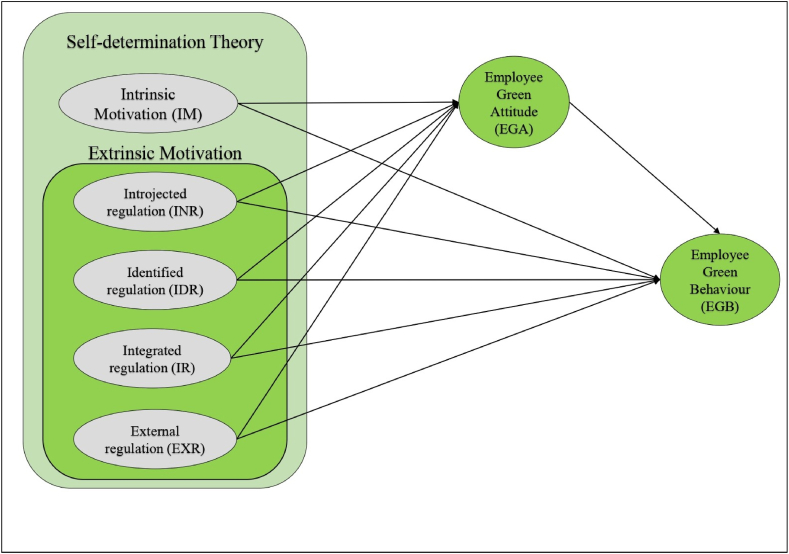
The conceptual framework ; **Source**: The author (2022)

A literature review suggests that an individual's ideology and personal concerns, such as ethical standards, environmental consciousness, and religiosity, are reflected in an employee's green behaviour, which promotes green culture, creativity, output, and engagement [36]. Depending on earlier literature, a descriptive outline for the variables, the study framework was constructed with five independent variables: intrinsic motivation, introjected regulation, identified regulation, integrated regulation, and external regulation (see Fig. 1). Employee green attitude is mediating variable, and employee green behaviour is the dependent variable.

### 3.1 Employee green attitude (EGA)

A practical or judgmental reaction to opinions is known as an attitude [37]. Research on green behaviour shows a link between proactive sustainable attitudes and proactive environmental behaviours, which is a critical hint in the arena [38]. Employee green behaviour is driven by internal factors such as a positive attitude toward the environment [39]. Figuring out how to promote EGB among personnel who do not have highly pro-environmental attitudes and beliefs is a crucial challenge for practitioners. The self-determination by which individual employees' green behaviour is influenced has yet to be empirically tested. Behavioural persistence may be an intermediary between attitude and behaviour [40]. Based on earlier research, this study argues that positive attitudes are associated with an increase in employee green behaviour and offer the subsequent hypothesis.

H1 Favourable employee green attitude is positively correlated with improved employee green behaviour.

### 3.2 Intrinsic Motivation(IM)

Depending on the values people may emphasize, different typologies of IM may stimulate pro-environmental behaviour [19]. Conceivably, employees may become more engaged in proactive green behaviour due to the satisfaction and enjoyment of doing so [34]. Employees may experience this satisfaction from their intrinsic desire for green creativity and their incentive to maintain a green image, which may encourage positive emotions [41]. Therefore, employees are self-motivated to connect in sustainable behaviour since they have an innate interest in safeguarding both their satisfaction and the well-being of the environment. The following hypotheses are based on the assumption that IM can favourably affect green attitudes and behaviour.

H2a Increased intrinsic motivation is correlated with favourable employee green attitude.

H2b Increased intrinsic motivation is correlated with favourable employee green behaviour.

### 3.3 Extrinsic motivation

In their work, Ryan and Deci examined self-regulation, which explores how individuals transform social values and extrinsic circumstances into internalized principles and self-generated motivations [23]. The desire for behaviour that results in the growth of external self-esteem indicators is called extrinsic motivation. Adults only sometimes engage in activities for fun (or intrinsic motivation). Instead, most actions are taken to get external rewards, such as improved social standing or financial gain [42]. Employee green behaviour may be more prevalent in firms when supportive leadership is an extrinsic motivational factor [43]. Bhutto et al. found a meaningful positive connection between employee adoption and a desire to show themselves positively in society by engaging in green behaviour that gives employees a more upbeat outlook on life [44]. Similar conclusions were reached by Abbas and Dogan, who concluded that green culture and socially responsible activities by the enterprise significantly alter employees' green behaviour [45]. Thus, positive correlations between employee green behaviour, employee green attitude, and the four types of extrinsic motivation (i.e., INR, IDR, IR, and EXR) are hypothesized.

### 3.4 Introjected regulation(INR)

Behaviours are performed as part of introjected regulation, an extrinsic motivation, to avert guilt or distress or to acquire ego boosts like pride [23]. Introjected regulation has been characterized as a minimal internal motivation that promotes people to assume the unspoken results of engaging in or refraining from specific conduct [42]. Prior research has shown increasing social pressures for green behaviour due to environmental activism, environmental protection concerns, and stakeholder demand [46,47]. Introjected regulation is positively connected to exerting more attempts, other than related to feeling more anxious and dealing with inferiority when failing [23]. Therefore, it is hypothesized that employees with greater INR will exhibit more positive attitudes and behaviours.

H3a Increased introjected regulation is correlated with favourable employee green attitude.

H3b Increased introjected regulation is correlated with favourable employee green behaviour.

### 3.5 Identified regulation(IDR)

Identified regulation is an independent type of extrinsic motivation. Identified regulations have been completely integrated into the self, which implies they have been reviewed and aligned with the individual's values and wants [23]. Identified regulation enables the achievement of critical goals and is motivated by personal beliefs and priorities. Due to identified motivation, employees who engage in PEBs do so because they care about protecting the environment [48]. Highly morally reflective employees frequently act in a manner that is consistent with their moral reasons [49]. Thus, it is assumed that workforces with higher IDR will have more positive attitudes and behaviours.

H4a Increased identified regulation is correlated with favourable employee green attitude.

H4b Increased identified regulation is correlated with favourable employee green behaviour.

### 3.6 Integrated regulation (IR)

Integrated regulation helps people adopt a particular behaviour and feel more self-aware and autonomous [50]. Integrated regulation involves engaging in behaviours consistent with multiple goals and values [51]. IR displays an employee's aspiration to engage in a precise action because they believe it will help them create a stronger sense of self [23]. Integrated regulation provides the most independent extrinsic motivation, expressing motives for actions fundamental to a person's identity [52]. Employee values are positively associated with environmental views related to perceived corporate citizenship. This kind of ownership significantly impacts corporate responsibility, employee values, and their views toward the environment [53]. Thus, employees with an advanced level of integrated regulation are more likely to employ green behaviour due to the engagement of their aligned personal beliefs and aspirations. These discussions suggested that IR should be favourably related to employee attitudes and green behaviour.

H5a Increased integrated regulation is correlated with favourable employee green attitude.

H5b Increased integrated regulation is correlated with favourable employee green behaviour.

### 3.7 External regulation (EXR)

External regulation (EXR) lacks internalized or autonomous behaviour and is governed by external influences [42]. Motives or controlled pressure from an external force that is not one's own can drive people [54]. External inspiration and PEBs have an affirmative association when corporate leadership is highly focused on sustainability [48]. Administrative stress on environmental protection and preference from consumers & market both positively impact green creativity and learning, which is linked to green innovation [55]. Externally driven employees may attempt to meet employment criteria, generate income and incentives, and confront or cease sanctions or the threat of punishment [23,48]. If employees' proactive green behaviour is adequately acknowledged and rewarded, it will be a new norm at the workplace [56]. Employee empowerment, performance evaluation, and training and development based on sustainability significantly influenced OCBE [31]. Thus, these pressures or advantages may serve as constructive external forces that encourage the development of stronger pro-green organizational attitudes and higher levels of green behaviour. The hypotheses listed below are proposed in light of these arguments.

H6a Increased external regulation is correlated with favourable employee green attitude.

H6b Increased external regulation is correlated with favourable employee green behaviour.

## 4 Research method and data

### 4.1 Sampling method, sample size, data collection and data analysis

Since this survey-based empirical research is interested in looking at associations between EGM (IV), EGB (DV), and EGA (MV) at a precise moment in time, it employs a cross-sectional methodology. The study's population includes around 2000 executives and managers at various levels working for private financial institutions/banks in Chattogram, Bangladesh. The sample is obtained from a non-normally distributed population. To uphold the 10:1 subject-to-item ratio, as Randall and Gibson recommended, studies must have at least 260 responders [57]. Between January and June 2022, 450 email addresses were sent questionnaires, and 377 responded—324 of which were acceptable (return rate 72%). The sample size is close to that used in studies on employee green behaviour and self-determination theory by other researchers [56]. To comprehend the background of the participants, data were used regarding gender, age, and education level. The average and median ages of the respondents were 35 and 40 years old, respectively. Male respondents made up the majority of the sample (70%). The preliminary analysis shows that most respondents have formal education, including master's degrees (56%) and undergraduate degrees (44%). It is worth mentioning that all participants provided verbal consent for the investigation.

A self-administered online-based questionnaire was used to collect data because of its adaptability and global reach [58]. Respondents had access to a google form containing the survey questionnaire via an electronic link. The questionnaire was divided into two segments. The first segment includes questions designed to capture demographic information from respondents, such as age, gender, organization name, and education level. For the second phase, a set of questions was prepared to measure green behaviour, attitude and motivation. The link was shared on whatsapp and via email. In addition, several reminders were issued to enhance the response rate. As a consequence of this approach, 324 responses may be utilized for further research from the total replies received.

This study used purposive sampling to select the respondents because this study has some particular aim. Purposive sampling, which is appropriate when an investigator uses the sample to adhere to specific, precise standards, is restricted to a small number of individuals who can deliver the specific necessary information as they are the only ones who have it or because they comply with specific requirements founded by investigators [59]. In the present research, respondents with appropriate awareness of environmental adulteration, resource scarcity, Etc., were purposively selected. The author assumes that executives and managers at private financial institutions know sustainability issues because they hold graduate degrees. In addition, they participate in various policy-making activities related to environmental compliance, green finance policy from the central bank, Etc. These are the primary intervention behind purposive sampling to pick executives and managers for the study.

Structural equation modeling (SEM) is usually used with a complex model containing many constructs, variables, indicators, and multiple relationships. There are two techniques for employing SEM: covariance-based techniques (CB-SEM) and variance-based partial least squares (PLS-SEM) [60]. For theory development and forecasting, PLS-based SEM is suitable at the exploratory stage. The study of formative and reflective relationships is possible with PLS-based SEM. PLS-SEM is regarded as the finest substitute for CB-SEM [61]. PLS-SEM, which differs from CB-SEM in that it is based on total variance, is a helpful technique for exploratory and confirmatory research [62]. PLS-SEM was used to carry out the two-step technique of Anderson and Gerbing for data analysis [63].

Confirmatory factor analysis (CFA) was executed initially to assess the measurement model, followed by the reliability and validity of the selected study measures (See Table 4). And then, the structural equation model was evaluated to investigate the proposed hypotheses. The descriptive statistics used in this study were chosen to present data frequencies for screening and assess data distribution normality with skewness and kurtosis measures and graphical portrayal. Kurtosis measures peakedness, and skewness measures symmetry. Normally distributed variables (mesokurtosis) have skewness and kurtosis values near zero [64].

The surveys were anonymous, consistent in appearance, and had no identifying questions on them. Respondents solely included self-reported measurements. Additionally, no records were kept of the respondents' private information, like the names, branches, or ranks. These measures were used to eliminate social desirability bias, a prevalent issue in this research [65].

Only cross-sectional data were utilized in this study. Cross-sectional research designs are often more efficient and less expensive than longitudinal designs, which follow the same set of participants over a more extended period. Since cross-sectional data capture a moment, they cannot be used to see how things have changed or to determine whether one thing caused another. Social desirability bias is also a possible limitation of the methodology, meaning that employees may exaggerate their degree of eco-friendly behaviour [65].

### 4.2 Measures and survey instrument design

The survey was divided into four sections: EGB-related measures, EGA-related measures, EGM-related measures, and demographics. Table 4 lists all constructs and the items adapted from early studies [23,66]. The EGB questionnaire is a 7-item scale that assesses an individual's behaviour towards the environment. The statements are scored on a 5-point Likert scale, with the options ranging from “strongly disagree” to “strongly agree”. Items such as “I ensure that air-conditioning is turned off when I am away from the workplace” and “I print and photocopy double-sided” are instances. Blok et al. conducted a study which revealed that the EGB questionnaire exhibited a high level of internal consistency, as evidenced by a cronbach alpha of 0.94 [24]. The instrument has been utilized in various investigations and has demonstrated acceptable levels of validity and reliability, as evidenced by prior research [67].

The 3-item EGA questionnaire measures attitudes towards environmentally friendly behaviour. The statements are rated on a Likert scale of 1–5, with 1 being “strongly disagree” and 5 being “strongly agree.” Examples are “I am in favour of green behaviour in the workplace” and “I think it is crucial to practice green behaviour at the workplace”. According to Blok et al.'s research, the EGA questionnaire demonstrated a high level of internal consistency, as evidenced by a Cronbach's alpha coefficient of 0.86 [24]. The scale has also been used in other studies and has shown reasonable validity and reliability [68].

Employee green motivation (EGM) is evaluated through a 16-item scale questionnaire comprising five distinct variables: IM, INR, IDR, IR, and EXR. The responses to the statements are also evaluated using a 5-point Likert scale, encompassing a range of options from “strongly disagree” to “strongly agree".

The IM questionnaire is a 3-subitem scale that assesses intrinsic motivation towards green behaviour. For example, “I enjoy using green practices to help safeguard the environment”. A study by Tremblay et al. [66] found that the IM questionnaire had a cronbach alpha of 0.77, representing high level of internal consistency. The INR questionnaire is a measurement tool consisting of three sub-items that evaluate the degree of introjected regulation in relation to environmentally-friendly behaviour. The following is an exemplary item: “I would feel proud of me if I do something to benefit the environment”. Tremblay et al. conducted a study which revealed that the INR questionnaire exhibited noteworthy internal consistency, as evidenced by a cronbach alpha of 0.71.

The 3-item IDR questionnaire assesses identified regulations towards green behaviour. “My desire for a greener earth and a sustainable generation will be fulfilled if I practice environmentally friendly conduct”, for instance. Tremblay et al. [66] discovered a cronbach alpha of 0.74 for the IDR questionnaire, indicating strong internal consistency. The IR questionnaire, a three-item measure, evaluates integrated regulation towards green behaviour. Example item: “My identity as a good citizen can be demonstrated by environment-friendly behaviour”. A study by Tremblay et al. [66] found that the IR questionnaire had a cronbach alpha of 0.84, indicating good internal consistency.

The EXR questionnaire consists of four sub-items that evaluate the degree of external regulation of environmentally conscious behaviour. An example item includes “Green behaviour helps me to avoid punishment”. According to research by Tremblay et al., the EXR questionnaire has been shown to have strong internal consistency, with a cronbach alpha of 0.81 [66]. The EGM questionnaire scale has also been used in other studies and has shown reasonable validity and reliability [69].

## 5 Results

### 5.1 Descriptive statistics

Responses for employee green behaviour ranged from 1.71 to 4.43, with a mean of 3.09 and a standard deviation of 0.59. The negligible skewness of −0.25 indicated that the distribution was skewed to the left. The bell curve was slightly platykurtic with a value of −0.89, demonstrating that it is likely to create fewer and less severe outliers than the normal distribution. These values differed marginally from the mean and have been presented in Table 1. The employee green attitude values ranged from a minimum of 2.00 to a maximum of 5.00, with a mean of 4.04 and a standard deviation of 0.65. The measured skewness was −0.72. With a measured value of 0.47, the curve was mildly leptokurtic. These results also departed slightly from the usual, as shown in Table 1.

**Table 1:** Descriptive statistics for sample population (N = 324).


	N	Minimum	Maximum	Mean	Std. Deviation	Skewness		Kurtosis	
	Statistic	Statistic	Statistic	Statistic	Statistic	Statistic	Std. Error	Statistic	Std. Error
EGB	324	1.71	4.43	3.09	0.59	−.25	.135	−.89	.270
EGA	324	2.00	5.00	4.04	0.65	−.72	.135	.47	.270
IM	324	2.33	5.00	3.61	0.46	−.29	.135	.32	.270
INR	324	2.00	5.00	3.47	0.62	.69	.135	.58	.270
IDR	324	1.67	5.00	2.82	0.68	1.32	.135	1.72	.270
IR	324	1.00	4.33	1.73	0.81	1.61	.135	2.02	.270
EXR	324	1.75	5.00	2.59	0.53	2.22	.135	6.28	.270
Valid N (listwise)	324								

Note: EGA = Employee Green Attitude, EGB = Employee Green Behaviour, EXR = External Regulation.

IDR= Identified Regulation, IM= Intrinsic Motivation, INR= Introjected Regulation, IR= Integrated Regulation.

A third set of descriptive statistics for intrinsic motivation was created. The response values ranged from 2.33 to 5.00, with a mean value of 3.61 and a standard deviation of 0.46. The measured skewness was −0.29, and the measured leptokurtic value of the curve was 0.32. These results also departed slightly from the normal, as shown in Table 1. A fourth set of descriptive statistics was generated for introjected regulation. The response values ranged from a minimum of 2.00 to a maximum of 5.00, with a mean of 3.47and a standard deviation of 0.62. The result of 0.69 for skewness indicated that the tail of the distribution was to the right, which extended to higher positive values. With an observed value of 0.58, the curve was mildly leptokurtic. These results also departed slightly from the usual, as shown in Table 1.

A fifth set of descriptive statistics was produced for the identified regulation. The response values ranged from a minimum of 1.67 to a maximum of 5.00, with a mean of 2.82 and a standard deviation of 0.68. The skewness, measured at 1.32, indicates that the tail was on the right side of the distribution, which was skewed towards more positive values, and the bell curve, measured at 1.72 was leptokurtic. These results also differed only slightly from the mean reported in Table 1. A sixth set of descriptive statistics was produced for integrated regulation. The response values ranged from a minimum value of 1.00 to a maximum value of 4.33, with a mean of 1.73 and a standard deviation of 0.81. The skewness was measured as 1.61, and the bell curve was leptokurtic with a value of 2.02. The final set of descriptive statistics was for external regulation. The response values ranged from 1.75 to 5.00, with a mean of 2.59 and a standard deviation of 0.53. A skewness of 2.22 shows that the tail was on the right side of the distribution, which extended towards higher positive values. The bell curve was leptokurtic with a reading of 6.28, indicating that the distribution had heavier tails than the normal distribution.

“Kolmogorov-Smirnov and Shapiro-Wilk” tests were used to determine whether the data distributions were normal. The “Kolmogorov-Smirnov” test found that the data distribution for EGB's responses was not normal. This was in line with the “Shapiro-Wilk test” results, which found that the data distribution was significantly different from normal. EGA, IM, INR, IDR, IR, and EXR responses were tested similarly. Table 2 shows that both distributions varied significantly from normality. The population data does not have a normal distribution because all non-parametric statistical analyses were distribution-free. The sample was drawn from a population that is not normally distributed. This research used a purposive sampling method, and the data were non-parametric. So, lack of normality is not a problem because of non-parametric data.

**Table 2:** Tests of normality.


	Kolmogorov-Smirnov^a^			Shapiro-Wilk		
	Statistic	df	Sig.	Statistic	df	Sig.
EGB	.102	324	.000	.966	324	.000
EGA	.149	324	.000	.936	324	.000
IM	.202	324	.000	.940	324	.000
INR	.198	324	.000	.924	324	.000
IDR	.233	324	.000	.864	324	.000
IR	.255	324	.000	.775	324	.000
EXR	.270	324	.000	.748	324	.000

a. Lilliefors Significance Correction.

Note: EGA = Employee Green Attitude, EGB = Employee Green Behaviour, EXR = External Regulation.

IDR= Identified Regulation, IM= Intrinsic Motivation, INR= Introjected Regulation, IR= Integrated Regulation.

Harman's single factor test was used to evaluate common method bias by SPSS. According to the statistics, the study had no issues with common method bias because the studied items made up 16.60% of the variation as per Table 3, which was less than 50% [70].

**Table 3:** Common method bias (total variance explained).

Component	Initial Eigenvalues			Extraction Sums of Squared Loadings		
	Total	% of Variance	Cumulative %	Total	% of Variance	Cumulative %
1	4.32	16.60	16.60	4.32	16.60	16.60
2	2.63	10.11	26.71			
3	2.46	9.48	36.19			
4	2.31	8.88	45.07			
5	1.67	6.44	51.50			
6	1.31	5.05	56.56			
7	1.25	4.82	61.38			
8	1.04	3.99	65.36			
9	0.94	3.60	68.97			
10	0.87	3.34	72.30			
11	0.83	3.21	75.51			
12	0.79	3.04	78.55			
13	0.72	2.76	81.31			
14	0.67	2.58	83.89			
15	0.65	2.48	86.37			
16	0.56	2.15	88.52			
17	0.52	1.98	90.51			
18	0.42	1.62	92.12			
19	0.40	1.52	93.64			
20	0.36	1.38	95.03			
21	0.30	1.13	96.16			
22	0.27	1.04	97.20			
23	0.25	0.95	98.14			
24	0.22	0.86	99.00			
25	0.15	0.59	99.59			
26	0.11	0.41	100.00			
Extraction Method: Principal Component Analysis.						

### 5.2 The results of the reflective measurement model (validity and reliability)

The projected study model and hypotheses were tested using PLS-SEM, a statistical tool to analyze and assess the connection among the observed elements. Five independent variables—intrinsic motivation, introjected regulation, identified regulation, integrated regulation and external regulation—were used to conceptualize the research model. The EGA was a mediator between the EGM and the EGB. The structural and reflective measurement models were the two levels of analysis that makeup PLS-SEM.

The author examined the factor loadings to determine the internal consistency, and the results are presented in Table 4. As per Table 4, all factor loadings were within the suggested range of 0.70 except for EGB7 [71].

**Table 4:** Confirmatory factor analysis (CFA) – (Outer Loadings).

No.	Items of the Constructs	Outer Loadings
EGA1	I am in favor of green behaviour in the workplace	0.819
EGA2	I think that encouraging employees to practice green behaviour at work is a wonderful idea	0.838
EGA3	I think it's crucial to practice green behaviour at workplace	0.833
EGB1	I ensure that air-conditioning is turned off When I'm away from the workplace	0.763
EGB2	I print and photocopy double-sided	0.760
EGB3	I sustainably use water for drinking and cleaning (reduce water waste, reuse & recycle water when possible)	0.795
EGB4	I pay attention and preferences to environment and sustainability during the purchase goods or services	0.796
EGB5	I turn off my computer/notebook/devices when I depart the workplace for a long time	0.787
EGB6	I turn off the lights when I leave my office for a while, and when there is no one else.	0.793
EGB7	I recycle and reuse plastics	0.660
EXR1	I do green behaviour because it has high social and national benefits	0.788
EXR2	Green behaviour helps me to avoid punishment	0.788
EXR3	Green behaviour helps me to get reward	0.700
EXR4	I've to behave green due to team/social/institutional pressure	0.731
IDR1	I believe this is meaningful and important to practice environmentally friendly conduct	0.743
IDR2	My desire for a greener earth and a sustainable generation will be fulfilled if I practice environmentally friendly conduct	0.863
IDR3	My organization/Team/Colleagues/Society appreciate the efforts to work sustainably	0.794
IM1	I enjoy using green practices to help safeguard the environment	0.814
IM2	It is interesting to take actions that help the environment	0.714
IM3	It gives me inner satisfaction to do green behaviour	0.856
INR1	I'll repent it if I don't take action to protect the environment and coming generations	0.820
INR2	I'll be embarrassed about it If I do nothing to conserve the environment	0.894
INR3	I would feel proud of me if I do something to benefit the environment	0.884
IR1	My identity of a good citizen can be demonstrated by environment friendly behaviour	0.914
IR2	Green behaviour has been a part of my Art of living/life style	0.943
IR3	Green behaviour is a fundamental part of who I am. (e.g. ‘taking care of the environment is an integral part of my life’)	0.894

Note: EGA = Employee Green Attitude, EGB = Employee Green Behaviour, EXR = External Regulation.

IDR= Identified Regulation, IM= Intrinsic Motivation, INR= Introjected Regulation, IR= Integrated Regulation.

Then “convergent” and “discriminant validity” were evaluated in the reflective measurement model. Convergent validity is the degree to which variables of a definite construct “converge or share a large proportion of variance in common” [71]. To measure convergent validity, Hair et al. recommended calculating the composite reliability (CR) and average variance extracted (AVE) [71]. Table 5 shows that, as Hair et al. indicated, the CR and AVE values were greater than the cut-off points of 0.70 and 0.50, respectively. That supports the constructs' validity and convergent reliability.

**Table 5:** Construct reliability and validity.

	Cronbach's alpha	Composite reliability (rho_a)	Composite reliability (rho_c)	Average variance extracted (AVE)
External regulation	0.744	0.744	0.839	0.567
Employee green attitude	0.774	0.775	0.869	0.689
Employee green behaviuor	0.882	0.886	0.908	0.587
Identified regulation	0.725	0.758	0.843	0.643
Intrinsic motivation	0.714	0.737	0.838	0.635
Introjected regulation	0.834	0.838	0.900	0.751
Integrated regulation	0.905	0.909	0.941	0.841

Then, the discriminant validity was evaluated using two methods. At first, as per Fornell and Larcker, the average variance between each construct and its measurements ought to be higher than the variance between the construct and other constructs [72]. As the square root of the AVE (diagonal) was larger than the correlations (off-diagonal) for all constructs [72], the results pointed out that every construct had attained satisfactory 'discriminant validity’, as shown in Table 6.

**Table 6:** Discriminant validity using the Fornell–Larcker criterion.

	EGA	EGB	EXR	IDR	IM	INR	IR
EGA	0.830						
EGB	0.528	0.766					
EXR	0.330	0.299	0.753				
IDR	0.426	0.367	0.072	0.802			
IM	0.394	0.574	0.313	0.183	0.797		
INR	0.439	0.321	0.085	0.434	0.217	0.867	
IR	0.335	0.378	0.189	0.259	0.266	0.174	0.917

Note: EGA = Employee green attitude, EGB = Employee green behaviour, EXR = External regulation.

IDR= Identified regulation, IM= Intrinsic motivation, INR= Introjected regulation, IR= Integrated regulation.

After that, discriminant validity was evaluated using the Heterotrait-Monotrait ratio (HTMT) technique developed by Henseler et al. [61]. As all scores exceeded the HTMT criterion [61], as per Table 7, the outcome demonstrated that discriminant validity was attained. Additionally, the HTMT results demonstrated that the confidence interval did not include one for any of the constructs, supporting the discriminant validity.

**Table 7:** Discriminant Validity: Heterotrait–Monotrait ratio (HTMT).

	EXR	EGA	EGB	IDR	IM	INR	IR
EXR							
EGA	0.435						
EGB	0.361	0.633					
IDR	0.133	0.553	0.45				
IM	0.433	0.522	0.704	0.248			
INR	0.111	0.545	0.376	0.544	0.274		
IR	0.229	0.395	0.421	0.33	0.332	0.198	

Note: EGA = Employee green attitude, EGB = Employee green behaviour, EXR = External regulation.

IDR= Identified regulation, IM= Intrinsic motivation, INR= Introjected regulation, IR= Integrated regulation.

### 5.3 Assessment of the structural model

When assessing a structural model, it is crucial to assess collinearity issues first; a collinearity problem may exist when the VIF is five or lower as per Hair et al. [73] or following the stricter standards set by Diamantopoulos and Sigouw [74], 3.3 or lesser. According to this report, all inner VIFs for the independent variables were less than 5 and 3.3 (see Table 8); this demonstrated the absence of collinearity, as per Hair et al. [75].

**Table 8:** Collinearity statistics (VIF)-Inner model.

	EXR	Employee Green Attitude	Employee Green Behaviuor
EXR		1.124	1.193
Employee Green Attitude			1.667
Employee Green Behaviuor			
IDR		1.291	1.378
IM		1.205	1.269
INR		1.264	1.373
IR		1.148	1.181

Note: EXR = External regulation, IDR= Identified regulation, IM= Intrinsic motivation,INR= Introjected regulation, IR= Integrated regulation.

**Fig. 2:**
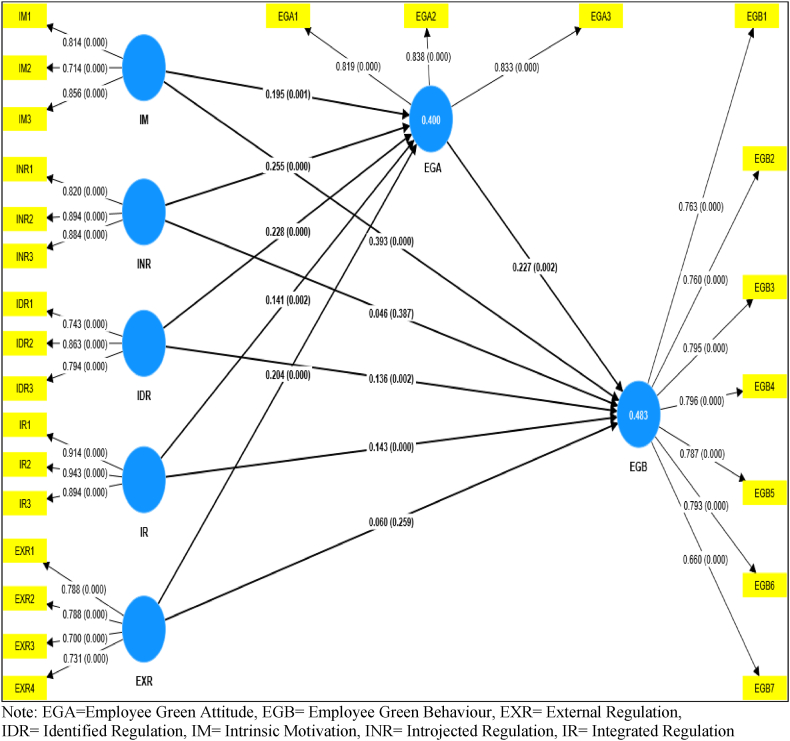
The structural model for self-determination, employee green attitude and employee green behaviour. **Source**: The author (2022), PLS-SEM (SMART-PLS4)

The relative weights of the exogenous constructs of intrinsic motivation (IM), introjected regulation (INR), identified regulation (IDR), integrated regulation (IR), and external regulation (EXR) in forecasting the endogenous construct of employees' green attitude (EGA) has been depicted in Table 9 and Fig. 2.

**Table 9:** Path coefficients (Direct effect) and hypothesis testing.

Path	Beta Coefficient (β)	Sample mean (M)	Standard deviation (STDEV)	T values	P values	CI-BC		Decision	Mediation Type
						2.50%	97.50%		
									
*EGA -> EGB*	0.227	0.217	0.073	3.122	0.002	0.096	0.379	Significant	Partial
*IM -> EGA*	0.195	0.191	0.057	3.454	0.001	0.085	0.304	Significant	Direct
*INR -> EGA*	0.255	0.257	0.046	5.529	0.000	0.161	0.344	Significant	Direct
*IDR -> EGA*	0.228	0.227	0.042	5.417	0.000	0.146	0.311	Significant	Direct
*IR -> EGA*	0.141	0.139	0.046	3.046	0.002	0.049	0.232	Significant	Direct
*EXR -> EGA*	0.204	0.209	0.052	3.939	0.000	0.098	0.301	Significant	Direct
*IM -> EGB*	0.393	0.396	0.056	6.961	0.000	0.274	0.494	Significant	Direct
*INR -> EGB*	0.046	0.052	0.053	0.865	0.387	−0.067	0.142	Insignificant	*No mediation*
*IDR -> EGB*	0.136	0.139	0.043	3.163	0.002	0.054	0.221	Significant	Direct
*IR -> EGB*	0.143	0.143	0.040	3.583	0.000	0.064	0.221	Significant	Direct
*EXR -> EGB*	0.060	0.063	0.054	1.129	0.259	−0.056	0.154	Insignificant	*No mediation*

Note: *** p-value <0.05, EGA = Employee Green Attitude, EGB = Employee Green Behaviour, EXR = External Regulation.

IDR= Identified Regulation, IM= Intrinsic Motivation, INR= Introjected Regulation, IR= Integrated Regulation.

CI-BC = Confidence interval bias corrected.

Source: PLS-SEM (SMART-PLS4).

### 5.4 Mediation analysis

The study used a sample of n = 324 respondents who completed a survey that included EGM, EGA, and EGB measures. The survey data were analyzed using Smart-PLS4 software to conduct a mediation analysis. The analysis included the following steps: 1) Descriptive statistics were computed for all variables to determine their means, standard deviations, and correlations. 2) The direct effect of EGM on EGB was estimated using a linear regression analysis. 3) The indirect effect of EGM on EGB through EGA was estimated using the bootstrap procedure with 5000 resamples to calculate bias-corrected confidence intervals (CI–BCs). 4) The total effect of EGM on EGB, which is the sum of the direct and indirect effects, was also estimated.

Table 9 represents the indirect effects of each independent variable (IM, INR, IDR, IR, and EXR) on the dependent variable (EGB) through the mediator variable EGA and the direct effects of the predictor variables on EGA and EGB.

The results indicated that all EGM variables except INR and EXR have a significant direct effect on EGB. The study has found a statistically significant direct effect of IM on EGB (β = 0.393, t = 6.961 > 1.96, p < 0.001), with a 97.5% confidence interval using bias-corrected bootstrapping (CI-BC) ranging from 0.274 to 0. 494.Conversely, the study has found that the relationship of INR on EGB is not statistically significant, as indicated by the beta coefficient of 0.046, t-value of 0.865 (which is less than the critical value of 1.96), p-value of 0.387, and confidence interval of [−0.067, 0.142]. The results of the statistical analysis demonstrate that the influence of IDR on EGB is also deemed to be statistically significant, as evidenced by the beta coefficient of 0.136, t-value of 3.163, which exceeds the critical value of 1.96, and a p-value of 0.002 with a 97.5% CI-BC ranging from 0.064 to 0.221. The study concludes that the relationship of EXR on EGB is not statistically significant (β = 0.060, t = 1.129 < 1.96, p = 0.259), with a 97.5% CI-BC interval of −0.056 to 0.154. Moreover, it can be observed that EGA has a noteworthy direct relationship (β = 0.227, t = 3.122 > 1.96, p = 0.002, CI-BC = [0.096, 0.379]) on EGB. This finding suggests that EGA mediates the association between the EGM variables and EGB; and has a partial direct influence on the outcome variable.

Furthermore, the author examined the indirect effects of the EGM variables (IM, INR, IDR, IR, and EXR) on the outcome variable EGB through the mediator variable EGA (see Table 10). The specific indirect effects of each EGM variable through EGA to EGB can be found in Table 10. The mediating role of EGA in the relationship between the independent variables and EGB exhibits significant indirect effects. The results indicate that the indirect relationship of IM on EGB through EGA is statistically significant (β = 0.044, t = 2.569 > 1.96, p = 0.010), with a 97.5% confidence interval using the bias-corrected bootstrap method ranging from 0.016 to 0.090. The results of the statistical analysis demonstrate that the influence of INR on EGB via EGA is significant, as evidenced by the considerable β value of 0.058 and t-value of 3.147 (p = 0.002). The confidence interval with a confidence level of 97.5%, which was computed using the bias-corrected and accelerated approach, spans from 0.027 to 0.099.The study findings indicate a significant indirect effect of IDR on EGB through EGA (β = 0.052, t = 2.477 > 1.96, p = 0.013), with a 97.5% CI-BC ranging from 0.019 to 0.104.The statistical analysis indicates that the relationship of IR on EGB is mediated by EGA, as evidenced by a significant indirect effect (β = 0.032, t = 2.144 > 1.96, p = 0.032) and a 97.5% CI-BC ranging from 0.010 to 0.073. Finally, the study results indicate that the relationship of EXR on EGB is mediated by EGA, as evidenced by a significant indirect effect (β = 0.046, t = 2.266 > 1.96, p = 0.023, 97.5% CI-BC = [0.014, 0.096]). All indirect effects were significant at the p < 0.05, indicating that EGA mediates the relationship between all EGM variables and EGB. This suggests that each of the five variables in EGM can influence EGB through their relationship with EGA. The magnitude of the indirect effects ranges from 0.032 to 0.058, suggesting that EGA has a moderate effect as a mediator.

**Table 10:** Specific Indirect effect and Total effect.

Path	Beta Coefficient (β)	Sample mean (M)	Standard deviation (STDEV)	T values	P values	CI-BC		Decision	Mediation Type
						2.50%	97.50%		
**Specific Indirect Effects**									
*IM -> EGA -> EGB*	0.044	0.041	0.017	2.569	0.010	0.016	0.090	Significant	Indirect
*INR -> EGA -> EGB*	0.058	0.055	0.018	3.147	0.002	0.027	0.099	Significant	Indirect
*IDR -> EGA -> EGB*	0.052	0.050	0.021	2.477	0.013	0.019	0.104	Significant	Indirect
*IR -> EGA -> EGB*	0.032	0.030	0.015	2.144	0.032	0.010	0.073	Significant	Indirect
*EXR -> EGA -> EGB*	0.046	0.046	0.020	2.266	0.023	0.014	0.096	Significant	Indirect
**Total effect**									
*IM -> EGB*	0.437	0.437	0.054	8.080	0.000	0.321	0.531	Significant	Direct
*INR -> EGB*	0.104	0.107	0.048	2.195	0.028	0.005	0.191	Significant	Direct
*IDR -> EGB*	0.188	0.189	0.043	4.423	0.000	0.105	0.272	Significant	Direct
*IR -> EGB*	0.175	0.173	0.040	4.435	0.000	0.098	0.252	Significant	Direct
*EXR -> EGB*	0.107	0.109	0.057	1.869	0.062	−0.015	0.210	Insignificant	No direct effect

Note: *** p-value <0.05, EGA = Employee Green Attitude, EGB = Employee Green Behaviour, EXR = External Regulation.

IDR= Identified Regulation, IM= Intrinsic Motivation, INR= Introjected Regulation, IR= Integrated Regulation.

Source: PLS-SEM (SMART-PLS4).

### 5.5 Explanatory power

The coefficient of determination R^2^ indicates how much variance in the dependent variable is explained by all of the independent variables connected to it, which gauge the model's forecast precision. The R^2^ of 0.40 indicates that intrinsic motivation, introjected regulation, identified regulation, integrated regulation, and external regulation can explain 40% of the total variation in employees' green attitudes. The R^2^ of 0.483 specifies that employees' green attitudes could explain 48.3% of the total variation in employee green behaviour.

The f^2^ values that were calculated to determine the effect size of each variable concerning the R^2^ were presented in Table 11. Cohen (1988) defined f^2^ values of 0.35, 0.15, and 0.02 as large, medium, and small effect sizes [76]. The f^2^ of 0.060 for EGA indicated a medium effect on the R^2^ for EGB. IM, INR, IDR, and EXR had moderate influences (f^2^: 0.053, 0.086, 0.067, 0.062) on R^2^ for EGA. Conversely, IR had a negligible effect (f^2^: 0.029) on R^2^ for EGA.

**Table 11:** Explanatory power.

Predictors	f^2^	R^2^	Q^2^
EGA-> EGB	0.060		
IM->EGA	0.053	0.400 (EGA)	0.371 (EGA)
INR->EGA	0.086		
IDR->EGA	0.067		
IR - >EGA	0.029		
EXR - >EGA	0.062		
IM - > EGB	0.235	0.483 (EGB)	0.480 (EGB)
INR - > EGB	0.003		
IDR - > EGB	0.026		
IR - >EGB	0.034		
EXR - >EGB	0.006		

Note: EGA = Employee Green Attitude, EGB = Employee Green Behaviour, EXR = External Regulation.

IDR= Identified Regulation, IM= Intrinsic Motivation, INR= Introjected Regulation, IR= Integrated Regulation.

Source: PLS-SEM (SMART-PLS4).

On the other hand, the f^2^ of 0.235 for IM indicated that it moderately affected the R^2^ for EGB. In opposition, the f^2^ of 0.026 and 0.034 for IDR and IR, respectively, indicated that they had a minimal effect on the R^2^ for EGB. The f^2^ of 0.003 and 0.006 for INR and EXR indicated that they did not affect the R^2^ for EGB.

Finally, the Q^2^ values of 0.371 and 0.480 for EGA and EGB, respectively, as shown in Table 11, are more than zero, indicating that the observed values were well reconstructed and that the model has predictive relevance as per Fornell and Cha [77].

## 6 Discussion

The relationship between EGA and EGB was examined in the first hypothesis (H1) and was found to be significant. Such a result corresponds to Tian et al., who described that a pro-environmental attitude helps to predict independent and monitored motivations, which helps to forecast necessary and spontaneous EGB [17]. The results are also consistent with those of Dodds et al. who concluded that intrinsic motivations are the driving force for sustainability management in festivals and are crucial for the overall integration of sustainability [35]. According to the significant association, employees' propensity to embrace positive attitudes towards green behaviour may help safeguard and sustain the environment. Green behaviour may be influenced by elements that foster a favourable attitude.

The next pair of hypotheses investigated how IM affected EGA (H2a) and EGB (H2b). The H2a and H2b results conclude that intrinsic motivational reasons are connected to the emergence of pro-environmental employee attitudes and green behaviours. This implies that employees' positive attitudes towards the environment may be attributed to their perceptions that adopting green behaviours might help to advance sustainability and protect the environment. These findings are also consistent with De Groot and Steg's [21]. The findings align with earlier studies by Sabbir and Taufique, which contend that IM, or inner motives, may increase a person's propensity to take actions helpful for the environment [32]. Such an outcome suggests that employees are more persuaded to adopt affirmative attitudes about sustainability and naturally enjoy engaging in activities that preserve and protect the environment.

This study has indicated a substantial link between INR and EGA (H3a). This suggests that attitudes toward the environment are influenced by externalized reasons linked to social influence or knowledge, such as guilt and disgrace. These results are consistent with past studies of Liao et al. and De Groot et al. [21,47]. In contrast, the results show that the link between INR and EGB (H3b) exhibits externalized motivations unrelated to workplace green behaviour, which is quite interesting. This is in opposition to De Groot et al. and Dodds et al. [21,35], who claimed that individuals' self-determined motivation orientation and ideologies influence proactive environmental intents. Employees may be influenced by extrinsic elements that produce favourable attitudes but not natural behaviour, which could explain inconsistent findings about introjected regulations. The results suggest that more than a positive outlook on sustainability is essential but may be needed for promoting environmentally responsible behaviour. When no one is watching, employees' actions could change. Another possible inference is that employees' outward displays of environmental activism and attitudes camouflage a lack of commitment to green practices in their personal lives. External-oriented motivations, such as social norms or desirability, increasingly induce Bangladeshi employees to exhibit a green attitude.

The subsequent hypotheses investigated the relationship between IDR and EGA (H4a), IDR and EGB (H4b). The extrinsic motivation of this kind is more self-reliant than other motivational factors. The study's findings support hypotheses H3a and H3b, suggesting that personal values encourage employees to instill a constructive attitude to green practices. It is in accordance with a previous investigation by Graves et al. showing that individual priorities and values can affect environmental behaviour since employees care about protecting the environment [48]. The subsequent hypotheses focused on the relationship between IR and EGA (H5a) and EGB (H5b). The study has found evidence to support hypotheses H5a and H5b, which point to the potential for employees' good citizenship intentions, preference for a green lifestyle, self-identity, Etc., to encourage employees to adopt a positive attitude and behaviour toward the environment. As a result, it can be concluded that employees are influenced by IR-related factors, which is harmonious with the prediction put forth by Manninen et al., who posited that self-identity is a crucial predictor of pro-green behaviour [52].

The relationships between EXR, EGA, and EGB were investigated using hypotheses H6a and H6b, respectively. The relationships between EXR and EGA were statistically significant, conforming to the study by Sharmin et al. [31] piloted in Bangladesh, which revealed that sustainability-centric employee empowerment, performance appraisal, and training and development significantly improve OCBE. On the other hand, the relationships between EXR and EGB (H6b) were found insignificant. Similar to INR, this disagrees with the findings of De Groot and Steg [21]. Nevertheless, the findings also go against the findings of Kim et al., who concluded that external regulation substantially influences environmental concern, self-efficacy, and behaviour related to the promotion of green ideas [34]. According to the study's findings, external factors and incentives can help employees develop a positive attitude towards environmental protection. On the other hand, the insignificant outcome of H6b can be explained as external regulations on green practices that need to be revised to transform the employees' green attitude into green behaviour due to a lack of proper policy, support, implementation and monitoring.

The findings of the mediation analysis suggest that EGA partially mediates the relationship between EGM and EGB. This indicates that EGM may influence EGB through its relationship with MV (EGA), which affects EGB. The result is parallel with the conclusions of Tian et al. [17]. It similarly conforms to the results of Nawaz, who revealed that the green attitude of employees strongly mediates the connection between green leadership and green activities undertaken by the employees [68].

Additionally, the significant direct effect of EGM on EGB for all the five variables in EGM suggests that all five variables in EGM are essential predictors of EGB. The direct effects are statistically significant, based on the associated p-values. The t-values and confidence intervals give further insight into the estimates' accuracy and dependability. As all the significant direct effects have T-values greater than 3, it indicates that the relationships are statistically significant. Furthermore, for confidence intervals for each significant direct effect, the range of values does not include zero, which further supports the conclusion that the relationships are statistically significant.

Larger β values indicate a stronger relationship, while smaller β values indicate a weaker relationship between the IV and DV. Looking at the β values, it is clear that IM-EGB has the most significant effect size (β = 0.393), followed by INR-EGA (β = 0.255) and IDR-EGA (β = 0.228). INR and IDR have the most substantial direct effects on EGM, with β values of 0.255 and 0.228, respectively. However, IR and EXR have weaker direct effects, with β values of 0.141 and 0.204, respectively. IM has a moderate direct effect with a β value of 0.195.

It is noteworthy that even after considering the mediating role of EGA, the direct effects of EGM on EGB remain significant. This suggests that in addition to the indirect relationship through EGA, EGM may influence EGB in other ways. The data shows that all predictor variables have significant direct effects on EGA, with beta coefficients ranging from 0.141 for IR to 0.255 for INR, except for INR (0.046) and EXR (0.060), which have a non-significant direct effect. In terms of the specific indirect effects, all predictor variables have significant indirect effects on EGB through EGA, with beta coefficients ranging from 0.032 for IR to 0.058 for INR. Overall, these results suggest that the EGM have both direct and indirect effects on the EGB and that the EGA mediates some of these effects. However, the size and direction of these effects vary depending on the independent variable (IM, INR, IDR, IR, and EXR) and the type of effect (direct or indirect).

The investigation shows the degree and direction of the mediated relationships, demonstrating that INR, IDR, and EXR may have potent indirect effects on EGB via the mediator EGA. Further investigation is required to completely comprehend the complexities of these relationships and account for potential confounding variables. According to these findings, the mediator variable EGA is crucial to the relationship between the predictor and outcome variables. INR and IDR have the most significant direct relationships on EGA, whereas INR and IDR have the most specific indirect effects on EGB via EGA. The non-significant direct influence of INR and EXR on EGB shows that these variables may only indirectly affect the outcome variable mediated by EGA.

## 7 Conclusion and implications

### 7.1 Research conclusion

This study aimed to examine the relationships between employees' attitudes, motivational dynamics, and green behaviour in the workplace in the context of Bangladeshi financial institutions. The study draws on a research model based on self-determination theory and tests it with 324 workers using a structural equation model. According to the findings, employee green attitude can mediate all five direct links between one independent variable (intrinsic motivation) and employee green behaviour and between four categories of extrinsic motivation and employee green behaviour. The findings suggest that intrinsic motivation, introjected regulation, identified regulation, integrated regulation, and external regulation all play a role in determining an employee's attitude toward sustainable behaviour, with some exceptions. The results also suggest that the relationship between introjected and external regulation of employees' pro-environmental behaviour is limited. Hence, emotions such as repentance, embarrassment, and pride have little association with the green behaviour of employees. While these feelings may encourage temporary behaviour adjustments, they may not necessarily develop a genuine commitment to sustainability. At the same time, external regulations may set standards, but they may need help to alter behaviour successfully. Creating a culture of sustainability requires employee participation and objective ownership. Individual willingness and commitment to make sustainable decisions are necessary for success. These findings have far-reaching implications for the international community.

Several nations and organizations worldwide have established sustainability legislation and efforts, and it is essential to comprehend the limitations of such measures. The study's findings suggest that more than a top-down approach to sustainability may be needed to make a real difference. Instead, a more comprehensive strategy is required that promotes employee engagement, goal ownership, and a culture of sustainability. This strategy applies to any nation or group that seeks to promote sustainability and foster a more environmentally conscious society. In addition, the findings emphasized the significance of individual accountability in creating a sustainable future. Irrespective of any external constraints or rewards, it is crucial for individuals to find intrinsic motivation and commitment in embracing sustainable choices and integrating them into their daily lives. This message applies to individuals worldwide and can be utilized in both personal and professional settings. Ultimately, sustainable methods must be integrated into daily activities. Global efforts should have been made to safeguard the economy and ecology for coming generations, slow environmental deterioration and promote sustainable growth. Promoting green practices by human resource managers, academics, and policymakers may influence employees' environmental attitudes. Such campaigns might persuade employees to adopt environmentally friendly behaviours.

### 7.2 Research implications

#### 7.2.1 Theoretical implications

This paper has accomplished a crucial theoretical advancement in the field of knowledge. The “Self-determination theory” was applied in the current study instead of the “Theory of Planned Behaviour”, which serves as the basis of the prior sustainability literature. The application of self-determination theory has provided critical new understandings concerning the association of personal characteristics, like intrinsic and extrinsic incentives, with environmentally friendly behaviour at the workplace. This study has added to the present theoretical understanding by emphasizing factors contributing to increasing environmental awareness, a pro-green attitude, and pro-green behaviour. Furthermore, these variables have been studied in the background of a rising economy like Bangladesh, which provides new insights into the behavioural scenario of such current significance of sustainable behaviour. For example, integrated regulation proposes that Bangladeshi employees could be motivated to engage in green behaviour more than other motivators, such as a desire to see themselves as responsible citizens, a preference for eco-friendly living, or their fundamental identity to nature. The insignificant mediation effects of introjected regulation and external regulation on employee green behaviour point to the need for further research on the mediation effects and moderating effects of additional factors that might influence employee green behaviour. Potential moderators may include elements like academic levels of employees, gender, top management support, employee religiosity Etc., that could clarify employee intentions for green behaviour more precisely.

#### 7.2.2 Practical implications

This study's conclusions have significant practical implications. The findings that external and introjected regulation has an inadequate association with employee green attitude have significant implications for managers attempting to promote sustainability within their firms. Initially, the findings point out the significance of fostering a culture of sustainability inside the firm, which involves employees' active participation and engagement. Moreover, the findings emphasize the significance of an individual's willingness and commitment to incorporate sustainable choices and behaviours into daily living. This gives managers a roadmap for promoting sustainability within their organizations that goes beyond compliance with rules and focuses instead on fostering a sustainability culture driven by employee involvement and dedication. Human resource managers and policymakers should concentrate on informing employees regarding the advantages and favourable social consequences of adopting green culture to stimulate increased green behaviour. Specific initiatives may proactively address fundamental emotional needs and individual objectives that inspire or persuade employee motivation, like the urge to protect one's environment.

Additionally, extrinsic returns like boosting people's self-respect through social standing (or positive sanctions) should be emphasized in campaigns, appealing to employees' need for social acceptance. Policymakers might focus on tying environmentally friendly behaviour to a sense of obligation and self-actualization. In turn, the employee may value the willingness to adopt sustainable behaviour. This may influence an employee's intrinsic motivation, integrated motivation, and sense of environmental champion.

### 7.3 Limitations and future research

First, although high levels of introjected regulation correlate with enhanced green behaviour, researchers found that such social pressure may be affected by social desirability bias, and employees may report higher levels of environmentally friendly activity, as indicated by Juvan and Dolnicar [78]. Social desirability is one of the most pervasive biases affecting the validity of experimental and survey research findings. Second, the current study was limited to personnel in large metropolitan areas of Bangladesh; its usefulness may be limited. The findings may change depending on the people residing outside of cities. Third, it was based on cross-sectional data, which limits our ability to draw causal inferences. Fourth, the research was conducted with PLS-SEM; the limitation of PLS-SEM is that it may not be suitable for data with complex or nonlinear relationships among variables. Last but not least, the study has included only one mediator.

Future research might focus on the following topics. First, research might be done to determine whether and how age and gender disparities influence green attitudes and behaviour. Second, similar research is needed among personnel of various demographic backgrounds, ethnicity and religions. The employees of such ethnic groups may be more exposed to preserving the environment and more positively oriented towards green behaviour. Third, research is required on how performance management systems affect employee green attitudes and behaviours. Future research should also evaluate organizational/governmental support for environmentally friendly behaviour at work and legal policies. Fourth, future research should investigate other potential mediators that may influence the relationship between employee green motivation and employee green behaviour. Fifth, future research should use longitudinal data to examine the causal relationships between employee green motivation, employee green attitude and employee green behaviour. Lastly, as a Muslim-majority country, an environmental campaign concentrating on religiosity could further induce employees' green behaviour by emphasizing the extrinsic motivation of green behaviour, such as reducing waste is a holy-religious responsibility.

## Author contribution statement

Abdullah Mohammad Ahshanul Mamun: Conceived and designed the experiments; Performed the experiments; Analyzed and interpreted the data; Contributed reagents, materials, analysis tools or data; Wrote the paper.

## Data availability statement

Data will be made available on request.

## Additional information

Supplementary content related to this article has been published online at [URL].

## Declaration of competing interest

The authors declare that they have no known competing financial interests or personal relationships that could have appeared to influence the work reported in this paper.

## References

[1] Aftab J., Abid N., Cucari N., Savastano M. (2022 Aug 19). Green human resource management and environmental performance: the role of green innovation and environmental strategy in a developing country. Bus. Strat. Environ..

[2] Stern P.C. (2000). New environmental theories: toward a coherent theory of environmentally significant behavior. J. Soc. Issues.

[3] Unsworth K.L., Dmitrieva A., Adriasola E. (2013 Feb). Changing behaviour: increasing the effectiveness of workplace interventions in creating pro‐environmental behaviour change. J. Organ. Behav..

[4] Norton T.A., Parker S.L., Zacher H., Ashkanasy N.M. (2015 Mar). Employee green behavior: a theoretical framework, multilevel review, and future research agenda. Organ. Environ..

[5] Starik M., Marcus A.A. (2000 Aug 1). Introduction to the special research forum on the management of organizations in the natural environment: a field emerging from multiple paths, with many challenges ahead. Acad. Manag. J..

[6] Savitz A. (2013 Oct 21).

[7] Haugh H.M., Talwar A. (2010 Sep). How do corporations embed sustainability across the organization?. Acad. Manag. Learn. Educ..

[8] Florea L., Cheung Y.H., Herndon N.C. (2013 May). For all good reasons: role of values in organizational sustainability. J. Bus. Ethics.

[9] Perrini F., Tencati A. (2006 Sep). Sustainability and stakeholder management: the need for new corporate performance evaluation and reporting systems. Bus. Strat. Environ..

[10] Jackson S.E., Ones D.S., Dilchert S. (2012 Jun 18).

[11] Ashkanasy N.M. (2003 Dec 31). Emotions in organizations: a multi-level perspective. InMulti-level issues in organizational behavior and strategy.

[12] Saifulina N., Carballo‐Penela A. (2017 Jul). Promoting sustainable development at an organizational level: an analysis of the drivers of workplace environmentally friendly behaviour of employees. Sustain. Dev..

[13] Xin Y., Senin A.B. (2022 Jan 31). Features of environmental sustainability concerning environmental regulations, green innovation and social distribution in China. Higher Education and Oriental Studies.

[14] Foster B., Muhammad Z., Yusliza M.Y., Faezah J.N., Johansyah M.D., Yong J.Y., Ul-Haque A., Saputra J., Ramayah T., Fawehinmi O. (2022 Apr 8). Determinants of pro-environmental behaviour in the workplace. Sustainability.

[15] Sabokro M., Masud M.M., Kayedian A. (2021 Sep 1). The effect of green human resources management on corporate social responsibility, green psychological climate and employees' green behavior. J. Clean. Prod..

[16] Pinzone M., Guerci M., Lettieri E., Huisingh D. (2019 Jul 20). Effects of ‘green’training on pro-environmental behaviors and job satisfaction: evidence from the Italian healthcare sector. J. Clean. Prod..

[17] Tian H., Zhang J., Li J. (2020 Mar). The relationship between pro-environmental attitude and employee green behavior: the role of motivational states and green work climate perceptions. Environ. Sci. Pollut. Control Ser..

[18] Fu L., Zhang Y., Bai Y. (2017 Nov 15). Pro-environmental awareness and behaviors on campus: evidence from Tianjin, China. Eurasia J. Math. Sci. Technol. Educ..

[19] Steg L., Lindenberg S., Keizer K. (2016 Jul 12). Intrinsic motivation, norms and environmental behaviour: the dynamics of overarching goals. International Review of Environmental and Resource Economics.

[20] Saeed B.B., Afsar B., Hafeez S., Khan I., Tahir M., Afridi M.A. (2019 Mar). Promoting employee's proenvironmental behavior through green human resource management practices. Corp. Soc. Responsib. Environ. Manag..

[21] De Groot J.I., Steg L. (2010 Dec 1). Relationships between value orientations, self-determined motivational types and pro-environmental behavioural intentions. J. Environ. Psychol..

[22] Deci E.L., Ryan R.M. (1985 Jun 1). The general causality orientations scale: self-determination in personality. J. Res. Pers..

[23] Ryan R.M., Deci E.L. (2000 Jan). Self-determination theory and the facilitation of intrinsic motivation, social development, and well-being. Am. Psychol..

[24] Blok V., Wesselink R., Studynka O., Kemp R. (2015 Nov 1). Encouraging sustainability in the workplace: a survey on the pro-environmental behaviour of university employees. J. Clean. Prod..

[25] Ansari N.Y., Farrukh M., Raza A. (2021 Jan). Green human resource management and employees pro‐environmental behaviours: examining the underlying mechanism. Corp. Soc. Responsib. Environ. Manag..

[26] Graves L.M., Sarkis J. (2018 Sep 20). The role of employees' leadership perceptions, values, and motivation in employees' pro-environmental behaviors. J. Clean. Prod..

[27] Islam M.M., Irfan M., Shahbaz M., Vo X.V. (2022 Jan 1). Renewable and non-renewable energy consumption in Bangladesh: the relative influencing profiles of economic factors, urbanization, physical infrastructure and institutional quality. Renew. Energy.

[28] Amin S.B., Chang Y., Khan F., Taghizadeh-Hesary F. (2022 Feb 1). Energy security and sustainable energy policy in Bangladesh: from the lens of 4As framework. Energy Pol..

[29] Haolader F.A., Khan S.H. (2022 Aug 5). In Recognizing Green Skills through Non-formal Learning: A Comparative Study in Asia.

[30] Sarker S.A., Wang S., Adnan K.M., Pooja P., Akhi K., Akter K. (2022 Jul 15). Renewable energy in Bangladesh: economic growth and policy perspectives. Journal of Science and Technology Policy Management.

[31] Sharmin S., Rahman M.H., Karim D.N. (2022 Mar 30). Green human resource management practices and organizational citizenship behaviour towards the environment in the banking sector in Bangladesh. Bangladesh Journal of Public Administration.

[32] Sabbir M.M., Taufique K.M. (2022 Jan). Sustainable employee green behavior in the workplace: integrating cognitive and non‐cognitive factors in corporate environmental policy. Bus. Strat. Environ..

[33] Lu H., Zou J., Chen H., Long R. (2020 Jun 10). Promotion or inhibition? Moral norms, anticipated emotion and employee's pro-environmental behavior. J. Clean. Prod..

[34] Kim M., Lee S.M. (2022 Feb 28). Drivers and interrelationships of three types of pro-environmental behaviors in the workplace. Int. J. Contemp. Hospit. Manag..

[35] Dodds R., Holmes M., Novotny M. (2022 Mar 4). Because I believe in it: examining intrinsic and extrinsic motivations for sustainability in festivals through self-determination theory. Tour. Recreat. Res..

[36] Fawehinmi O., Yusliza M.Y., Wan Kasim W.Z., Mohamad Z., Sofian Abdul Halim M.A. (2020 Dec). Exploring the interplay of green human resource management, employee green behavior, and personal moral norms. Sage Open.

[37] Ajzen I. (1991 Dec 1). The theory of planned behavior. Organ. Behav. Hum. Decis. Process..

[38] Ashraf M.S., Hou F., Kim W.G., Ahmad W., Ashraf R.U. (2020 Jan). Modeling tourists' visiting intentions toward ecofriendly destinations: implications for sustainable tourism operators. Bus. Strat. Environ..

[39] Bissing‐Olson M.J., Iyer A., Fielding K.S., Zacher H. (2013 Feb). Relationships between daily affect and pro‐environmental behavior at work: the moderating role of pro‐environmental attitude. J. Organ. Behav..

[40] Sultan P., Tarafder T., Pearson D., Henryks J. (2020 Apr 1). Intention-behaviour gap and perceived behavioural control-behaviour gap in theory of planned behaviour: moderating roles of communication, satisfaction and trust in organic food consumption. Food Qual. Prefer..

[41] Liu B., Xu J., Guo Y., Fu Y. (2022 Sep 17). How the perceived value of green creativity influences employees' green creativity: the dual promotion–prevention path. J. Sustain. Tourism.

[42] Wang W.T., Hou Y.P. (2015 Jan 1). Motivations of employees' knowledge sharing behaviors: a self-determination perspective. Inf. Organ..

[43] Li W., Bhutto T.A., Xuhui W., Maitlo Q., Zafar A.U., Bhutto N.A. (2020 May 10). Unlocking employees' green creativity: the effects of green transformational leadership, green intrinsic, and extrinsic motivation. J. Clean. Prod..

[44] Bhutto T.A., Farooq R., Talwar S., Awan U., Dhir A. (2021 Oct 3). Green inclusive leadership and green creativity in the tourism and hospitality sector: serial mediation of green psychological climate and work engagement. J. Sustain. Tourism.

[45] Abbas J., Dogan E. (2022 Aug). The impacts of organizational green culture and corporate social responsibility on employees' responsible behaviour towards the society. Environ. Sci. Pollut. Control Ser..

[46] Nguyen N.P., Adomako S. (2022 Jan). Stakeholder pressure for eco‐friendly practices, international orientation, and eco‐innovation: a study of small and medium‐sized enterprises in Vietnam. Corp. Soc. Responsib. Environ. Manag..

[47] Liao B., Li L., Yang Z. (2022 Jan). Perceived social green preference: the motivation mechanism of inducing green behaviour. Curr. Psychol..

[48] Graves L.M., Sarkis J., Zhu Q. (2013 Sep 1). How transformational leadership and employee motivation combine to predict employee proenvironmental behaviors in China. J. Environ. Psychol..

[49] Kim A., Kim Y., Han K., Jackson S.E., Ployhart R.E. (2017 May). Multilevel influences on voluntary workplace green behavior: individual differences, leader behavior, and coworker advocacy. J. Manag..

[50] Gillison F., Osborn M., Standage M., Skevington S. (2009 May 1). Exploring the experience of introjected regulation for exercise across gender in adolescence. Psychol. Sport Exerc..

[51] Ryan R.M., Deci E.L. (2017 Feb 14).

[52] Manninen M., Dishman R., Hwang Y., Magrum E., Deng Y., Yli-Piipari S. (2022 Sep). Self-determination theory based instructional interventions and motivational regulations in organized physical activity: a systematic review and multivariate meta-analysis. Psychol. Sport Exerc..

[53] Gabler C.B., Itani O.S., Agnihotri R. (2022 Jul 20). Activating corporate environmental ethics on the frontline: a natural resource-based view. J. Bus. Ethics.

[54] Persaud A., Schillo S.R. (2017 Feb 6). Purchasing organic products: role of social context and consumer innovativeness. Market. Intell. Plann..

[55] Cui R., Wang J. (2022 May). Shaping sustainable development: external environmental pressure, exploratory green learning, and radical green innovation. Corp. Soc. Responsib. Environ. Manag..

[56] Darvishmotevali M., Altinay L. (2022 Feb 1). Green HRM, environmental awareness and green behaviors: the moderating role of servant leadership. Tourism Manag..

[57] Randall D.M., Gibson A.M. (2012 Jun 12).

[58] Evans J.R., Mathur A. (2005 Apr 1). The value of online surveys. Internet Res..

[59] Blumberg B., Cooper D., Schindler P. (2014 Mar 16).

[60] Sarstedt M., Ringle C.M., Smith D., Reams R., Hair J.F. (2014 Mar 1). Partial least squares structural equation modeling (PLS-SEM): a useful tool for family business researchers. Journal of family business strategy.

[61] Henseler J., Ringle C.M., Sarstedt M. (2015 Jan). A new criterion for assessing discriminant validity in variance-based structural equation modeling. J. Acad. Market. Sci..

[62] Hair J., Hollingsworth C.L., Randolph A.B., Chong A.Y. (2017 Apr 10).

[63] Anderson J.C., Gerbing D.W. (1988 May). Structural equation modeling in practice: a review and recommended two-step approach. Psychol. Bull..

[64] Meyers L.S., Gamst G., Guarino A.J. (2016 Oct 28).

[65] King M.F., Bruner G.C. (2000 Feb). Social desirability bias: a neglected aspect of validity testing. Psychol. Market..

[66] Tremblay M.A., Blanchard C.M., Taylor S., Pelletier L.G., Villeneuve M. (2009 Oct). Work extrinsic and intrinsic motivation scale: its value for organizational psychology research. Canadian Journal of Behavioural Science/Revue canadienne des sciences du comportement.

[67] Ahmad I., Ullah K., Khan A. (2022 Oct 28). The impact of green HRM on green creativity: mediating role of pro-environmental behaviors and moderating role of ethical leadership style. Int. J. Hum. Resour. Manag..

[68] Nawaz Khan A. (2022 Mar 4). Is green leadership associated with employees' green behavior? Role of green human resource management. J. Environ. Plann. Manag..

[69] Xu C. (2022 Apr). Work motivation in the public service: a scale development based on the self-determination theory. Sage Open.

[70] Podsakoff P.M., MacKenzie S.B., Lee J.Y., Podsakoff N.P. (2003 Oct). Common method biases in behavioral research: a critical review of the literature and recommended remedies. J. Appl. Psychol..

[71] Hair J.F., Hult G.T., Ringle C.M., Sarstedt M. (2021 Jun 30).

[72] Fornell C., Larcker D.F. (1981 Feb). Evaluating structural equation models with unobservable variables and measurement error. J. Market. Res..

[73] Hair J.F., Ringle C.M., Sarstedt M. (2011 Apr 1). PLS-SEM: indeed a silver bullet. J. Market. Theor. Pract..

[74] Diamantopoulos A., Siguaw J.A. (2006 Dec). Formative versus reflective indicators in organizational measure development: a comparison and empirical illustration. Br. J. Manag..

[75] Hair J.F., Risher J.J., Sarstedt M., Ringle C.M. (2019 Jan 14). When to use and how to report the results of PLS-SEM. Eur. Bus. Rev..

[76] Cohen J. (1988).

[77] Fornell C., Cha J., Bagozzi R. (1994). Advanced Methods of Market Research.

[78] Juvan E., Dolnicar S. (2016 Jul 1). Measuring environmentally sustainable tourist behaviour. Ann. Tourism Res..

