# Supplementary material for: Motivating green behaviour in Bangladeshi employees: Self-determination theory application
Source: Heliyon. 2023 Jul 13;9(7):e18155. doi: 10.1016/j.heliyon.2023.e18155 (PMC10368830; doi:10.1016/j.heliyon.2023.e18155)
Supplement: Multimedia component 1 [file mmc1.doc]

**DEMOGRAPHIC INFORMATION [Please tick (√) the appropriate box]**

| Name | : | **No Need Please to keep anonymity** | | | | |
| --- | --- | --- | --- | --- | --- | --- |
| Name of Organization | : |  | | | | |
| Gender | 1. | Male | 2. | Female |  |  |
| Age | 1. | 16-25 years | 2. | 26-35 years | 3. | 36-45 years |
|  | 4. | 46-55 years | 5. | 56 years and above | | |
| Education | 1. | Secondary (SSC) | 2. | HSC/Diploma | 3. | Bachelors degree |
|  | 4. | Master degree | 5. | PhD | 6. | Others |

| **Employee Green Behavior (EGB)** | | | | | | | | | |
| --- | --- | --- | --- | --- | --- | --- | --- | --- | --- |
|  |  | | | **Strongly Disagree**  **(1)** | | **Disagree**  **(2)** | **Neutral (3)** | **Agree**  **(4)** | **(Strongly Agree**  **(5)** |
| Q1 | I make sure that *air-conditioning* is switched off when I am not in the office | | | 1 | | 2 | 3 | 4 | 5 |
| Q2 | I *print and photocopy* double-sided | | | 1 | | 2 | 3 | 4 | 5 |
| Q3 | I sustainably *use water* for drinking and cleaning (reduce water waste, reuse & recycle water when possible) | | | 1 | | 2 | 3 | 4 | 5 |
| Q4 | I pay attention and preferences to environment and sustainability during the *purchase goods or services* | | | 1 | | 2 | 3 | 4 | 5 |
| Q5 | I switch off my *computer/notebook/devices* when I leave my office for a considerable period. | | | 1 | | 2 | 3 | 4 | 5 |
| Q6 | I switch off the *lights* when I leave my office for a considerable period, and when there is no one else. | | | 1 | | 2 | 3 | 4 | 5 |
| Q7 | I recycle and reuse plastics | | | 1 | | 2 | 3 | 4 | 5 |
| **Employee Green Attitude (EGA)** | | | | | | | | | |
| Q8 | | I am *in favor* of green behavior in the workplace | 1 | | 2 | | 3 | 4 | 5 |
| Q9 | | I think *it's a good idea* for employee to do green behavior in the workplace | 1 | | 2 | | 3 | 4 | 5 |
| Q10 | | Green behavior in the workplace *is important to me.* | 1 | | 2 | | 3 | 4 | 5 |
| **Intrinsic Motivation (IM)** | | | | | | | | | |
| Q11 | | It is my *pleasure to contribute* to protecting the environment by Green behavior | 1 | | 2 | | 3 | 4 | 5 |
| Q12 | | It is *interesting* to act in a way that benefits the environment | 1 | | 2 | | 3 | 4 | 5 |
| Q13 | | It gives me *inner satisfaction* to do green behavior | 1 | | 2 | | 3 | 4 | 5 |
| **Introjected regulation (INR)** | | | | | | | | | |
| Q14 | | I will *regret* it if I am not doing something for the environment and future generations | 1 | | 2 | | 3 | 4 | 5 |
| Q15 | | I will *feel ashamed* of myself if I doing nothing to help the environment | 1 | | 2 | | 3 | 4 | 5 |
| Q16 | | I would *feel proud* of myself if I do something to help the environment | 1 | | 2 | | 3 | 4 | 5 |
| **Identified Regulation (IDR)** | | | | | | | | | |
| Q17 | | I believe this is *meaningful and important* to practice environmentally friendly conduct. | 1 | | 2 | | 3 | 4 | 5 |
| Q18 | | My *desire* for a greener earth and a sustainable generation will be fulfilled if I practice environmentally friendly conduct | 1 | | 2 | | 3 | 4 | 5 |
| Q19 | | My organization/ Team/ Colleagues/ Society *appreciate* the efforts to work sustainably | 1 | | 2 | | 3 | 4 | 5 |
| **Integrated regulation (IR)** | | | | | | | | | |
| Q20 | | My identity of a good citizen can be *demonstrated* by environment friendly behavior | 1 | | 2 | | 3 | 4 | 5 |
| Q21 | | Green behavior has been a part of my *Art of living /life style.* | 1 | | 2 | | 3 | 4 | 5 |
| Q22 | | Green Behavior is a fundamental part of *who I am.* (e.g. “taking care of the environment is *an integral part of my life*”) | 1 | | 2 | | 3 | 4 | 5 |
| **External Regulation (EXR)** | | | | | | | | | |
| Q23 | | I do green behavior because it has high *social and national benefits* | 1 | | 2 | | 3 | 4 | 5 |
| Q24 | | Green behavior help me to *avoid punishment* | 1 | | 2 | | 3 | 4 | 5 |
| Q25 | | Green behavior help me to *get reward* | 1 | | 2 | | 3 | 4 | 5 |
| Q26 | | I’ve to behave green due to *team/social/institutional pressure* | 1 | | 2 | | 3 | 4 | 5 |
